# Identity Theft Among Older Adults: Risk and Protective Factors

**DOI:** 10.1093/geroni/igaa057.100

**Published:** 2020-12-16

**Authors:** Marguerite DeLiema, Lynn Langton, David Burnes

**Affiliations:** 1 University of Minnesota, Twin Cities, Saint Paul, Minnesota, United States; 2 RTI International, Washington, District of Columbia, United States; 3 University of Toronto, Toronto, Ontario, Canada

## Abstract

Although financial exploitation and fraud targeting older adults have been the focus of increasing academic attention, research on identity theft among older adults is virtually nonexistent. Identity theft refers to an intentional, unauthorized transfer or use of a person’s identifying information for unlawful purposes (Federal Trade Commission 1998). Society’s growing reliance on technology to transfer and store private information has created increased opportunities for financial predators to access and misuse personal data. Results from the most recent Bureau of Justice Statistics’ Identity Theft Supplement show that nearly 1 in 10 adults aged 65 or older experienced identity theft in the past year, with financial losses totaling $2.5 billion. Given the high frequency and cost of identity theft among older Americans, more research is needed to guide prevention efforts and interventions that support recovery. This paper examines the risk factors, protective factors, costs, and consequences of identity theft victimization among older adults, focusing on differences between those aged 65-74 and those 75 or older. Findings suggest that the prevalence of identity theft is lower among those 75 or older (6.6% versus 10.3%), but those 75 or older experienced higher average losses per identity theft incident ($155 vs $96). Compared to those aged 65-74, a lower percentage of adults aged 75 or older engaged in online shopping, thereby reducing their risk of identity exposure (48% versus 24%). However, they were also less likely to engage in protective behaviors such as checking credit reports, changing passwords, checking account statements, and using security software.

